# Clozapine and all‐cause mortality in treatment‐resistant schizophrenia: a historical cohort study

**DOI:** 10.1111/acps.12989

**Published:** 2018-12-16

**Authors:** J. Cho, R. D. Hayes, A. Jewell, G. Kadra, H. Shetty, J. H. MacCabe, J. Downs

**Affiliations:** ^1^ Institute of Psychiatry Psychology and Neuroscience King's College London London UK; ^2^ NIHR Maudsley Biomedical Research Centre London UK; ^3^ South London and Maudsley NHS Foundation Trust London UK

**Keywords:** clozapine, mortality, treatment‐resistant, schizophrenia

## Abstract

**Objective:**

Large‐scale epidemiological studies have demonstrated a protective effect of clozapine on mortality in people with schizophrenia. Clozapine is reserved for use in patients with treatment‐resistant schizophrenia (TRS), but evidence of clozapine's effect on mortality exclusively within TRS samples is inconclusive. Hence, we aimed to investigate the effect of clozapine use on all‐cause mortality in TRS patients.

**Methods:**

A historical patient cohort sample of 2837 patients, who met criteria for TRS between 1 Jan 2008 and 1 Jan 2016, were selected from the South London and Maudsley NHS Foundation Trust (SLAM) electronic health records (EHR). The national Zaponex Treatment Access System (ZTAS) mandatory monitoring system linked to the SLAM EHR was used to distinguish which patients were initiated on clozapine (*n* = 1025). Cox proportional hazard models were used, adjusting for sociodemographics, clinical monitoring, mental and physical illness severity and functional status.

**Results:**

After controlling for potential confounders, the protective effect of clozapine on all‐cause mortality was significant (adjusted hazard ratio 0.61; 95% confidence interval 0.38–0.97; *P* = 0.04).

**Conclusions:**

Clozapine reduces the risk of mortality in patients who meet criteria for TRS. We provide further evidence that improving access to clozapine in TRS is likely to reduce the mortality gap in schizophrenia.

**Significant outcomes**

- Among patients with treatment‐resistant schizophrenia (TRS), clozapine users had a reduced mortality risk compared to patients who have not used clozapine, after taking account for differential baseline risks including psychopathology and functional status, and addressing potential impact of survival biases.

**Limitations**

- An automated approach was used to identify patients who meet TRS criteria and who have not been previously trialled on clozapine. This might lead to misclassification, possibly including non‐TRS patients in the analysis. This misclassification is more likely to be non‐differential.
- There is potential residual confounding by unmeasured confounders such as comorbid cardiovascular disease and smoking.

## Introduction

Patients with schizophrenia and other psychoses have approximately three times higher rates of all‐cause mortality and over ten times higher rates of suicide compared to general population 1.

A number of epidemiological studies have recently examined how pharmacological treatments may be associated with differences in survival in populations with psychoses. The effects of clozapine, an atypical antipsychotic, on mortality in patients with psychoses has received particular attention 2, 3, 4, 5, 6, 7, 8, 9. Despite the varied comparators and study populations, several large‐scale studies with long‐term follow‐up have reported consistently a protective effect of clozapine use on mortality 9. However, clozapine use is restricted as a third‐line treatment option in most countries, and only licensed for patients with treatment‐resistant schizophrenia (TRS), where first‐ and second‐line antipsychotic treatment trials have failed. Ahead of using clozapine, patients undergo clinical examinations including routine haematological screening to reduce the risk of the rare but severe side‐effect of agranulocytosis 10. In most developed countries including the United Kingdom, clozapine therapy can only be initiated when minimum absolute neutrophil counts are achieved, followed by weekly to monthly blood profile monitoring 11. Consequently, clozapine users are likely to clinically differ from non‐clozapine users. Moreover, patients who meet the definition of treatment resistance may be biologically distinct from those with treatment‐responsive schizophrenia 12, 13, 14. In line with this, two recent studies have investigated the effect of clozapine use on mortality in TRS 8, 15. The study using United States Medicaid data reported no significant mortality difference between those patients treated with clozapine and those without 15, whereas the Danish national registry study found an approximately twofold higher mortality in non‐clozapine users 8. Without further study, these contrasting findings from two different healthcare systems make it difficult to determine whether clozapine does or does not reduce the risk of death in patients with treatment‐resistant schizophrenia.

Many patients who meet criteria for treatment resistance either never receive clozapine treatment or at least experience considerable delay in receiving it 16, 17.These studies imply a hesitation on the part of clinicians and/or patients. Their findings suggest barriers to earlier adoption of clozapine might be due to concerns over severe side‐effects, and difficulties with adhering to strict initiation guidelines and schedule of intensive clinical monitoring. Patients with TRS, prior to clozapine initiation, generally have complex treatment regimes. In an earlier study, we found 14% of patients with TRS on antipsychotic polypharmacy and 33% receiving adjuvant psychotropic drugs such as antidepressants and benzodiazepines 18. These more complex treatment patterns are associated with greater harm than monotherapy regimes. Antipsychotic polypharmacy has been associated with increased risks of readmission 19, adverse events such as extrapyramidal side‐effects 20, metabolic syndrome 21 and mortality 22, 23. In terms of preventing potentially avoidable deaths, it is important to discern whether clozapine is superior to other TRS regimens in reducing mortality. If this effect is confirmed, there is a greater argument for helping patients with TRS start and sustain clozapine treatment.

### Aims of the study

The present historical cohort study investigates the effect of clozapine use on all‐cause mortality in treatment‐resistant schizophrenia patients in the United Kingdom. Using electronic mental health records from community and in‐patient care settings linked to clozapine national registry data, we aimed to address several of the methodological weaknesses identified by a recent systematic review of prior work examining clozapine use and mortality in schizophrenia 9. These include issues of residual bias through selecting non‐treatment‐resistant schizophrenia controls 24, performance bias through better access to care, survival bias 2 and measurement bias through exclusion of hospital‐based prescribing 25.

## Material and methods

### Data sources

#### Clinical record interactive search

The Clinical Record Interactive Search (CRIS) has provided an anonymized search system for the electronic health records (EHR) of the South London and Maudsley NHS Foundation Trust (SLAM) from 2007 onwards (25–27). SLAM provides mental health specialist services in four London boroughs (Lambeth, Southwark, Lewisham and Croydon), covering roughly 1.36 million of population. Using the Using the CRIS system, researchers can query structured and free‐text data of over 300 000 patients. These patient records are updated by contemporaneous national mortality information (irrespective of current treatment status in SLAM), and enhanced by linkages to external clinical database such as the national Zaponex Treatment Access System (ZTAS) clozapine register.

#### ZTAS register

To prevent clozapine users from agranulocytosis‐related deaths, several countries including the United Kingdom, the United States, Australia and Canada operate a national registry and monitoring system for clozapine users with blood test results. Clozapine patients treated within SLAM are enrolled on ZTAS (http://www.ztas.co.uk), which is one of the UK mandatory monitoring systems. In order for clinicians to prescribe clozapine, they must register clozapine users on the ZTAS system, with patients’ pretreatment blood test results (white blood cell, neutrophil, eosinophil and platelet counts). The reference blood test results are categorized as green (white blood cell, 3.5 × 10^9^/L; neutrophil 2.0 × 10^9^/L), amber (3.0–3.5; 1.5–1.9) and red (<3.0; <1.5). Patients with ‘green’ blood test results can be prescribed clozapine, within 10 days of this result. In this study, a linkage was performed with all patients whose last recorded pharmacy within the SLAM boroughs, and who had been registered on the ZTAS monitoring service between 1 Jan 2007 and 1 Jan 2016. These data were used to confirm the initiation date of clozapine users first identified from the CRIS database.

### Ethical considerations

The use of anonymized data from the CRIS database for mental health research was approved by Oxfordshire Research Ethics Committee C (08/H0606/71+5), and governance is provided for all projects and dissemination through a patient‐led oversight committee.

### Study subjects

**Figure 1:**
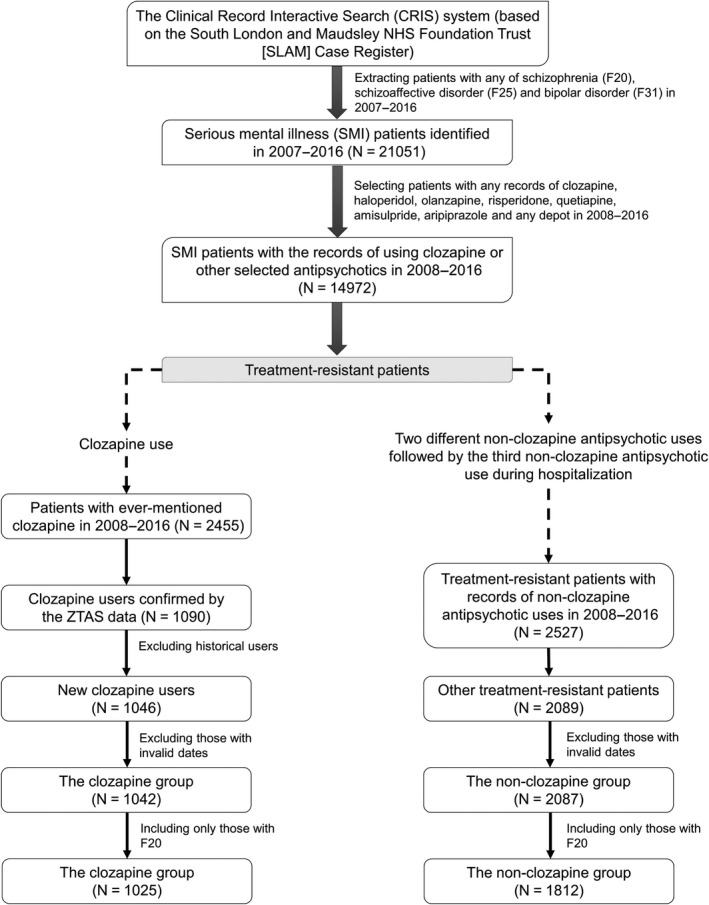
Selection of study subjects.

Please see Fig. 1 for a flow diagram describing the sample selection. We used a previously‐established approach 2 to identify the initial cohort which included all patients who received treatment from SLAM services between 1 Jan 2007 and 1 Jan 2016, who were aged 15 years and older, and had at least one recorded International Classification of Diseases version 10 (ICD‐10) diagnoses of schizophrenia (F20), schizoaffective disorder (F25) or bipolar disorder (F31) recorded during the observation period. From this cohort, we identified the six most commonly used oral antipsychotic medications: amisulpride, aripiprazole, haloperidol, olanzapine, risperidone, quetiapine and any British National Formulary listed long‐acting injectable or depot 11. We then identified those patients who had been prescribed any of these antipsychotics and/or clozapine. This yielded 14 972 subjects. Study subjects were identified using both structured and free‐text fields in the CRIS system. For the free‐text data, subjects’ information was extracted using a natural language processing (NLP) application which was developed using Generalized Architecture for Text Engineering (GATE). GATE enables researchers to extract data from clinical documents while considering the linguistic context. The performance of this NLP application for extracting medication prescribing has been previously validated and found to have good accuracy, for example identifying recorded clozapine use at a patient level with the precision and recall performance of 96% and 92% respectively 2.

### Identifying treatment resistance and clozapine exposure

Treatment‐resistant psychosis patients were defined as those who had ever been selected for clozapine treatment or those who had received a trial of at least three different antipsychotics, where the third novel antipsychotic was initiated during hospitalization between 1 Jan 2008 and 1 Jan 2016, according to SLAM EHR and ZTAS records. Among the initial cohort, 1042 subjects (the clozapine exposed group) had greater than one ‘green’ blood profiles on the ZTAS records. The earliest date was used as an indicative date for clozapine initiation. For the non‐clozapine group, 2089 patients met the definition of treatment resistance, with no evidence of clozapine use. After excluding 2 subjects with invalid dates, 2087 treatment‐resistant patients with non‐clozapine antipsychotic use were identified. Among the identified clozapine and non‐clozapine groups, we then restricted the sample to include only those who had received at least one ICD‐10 schizophrenia (F20) diagnoses (Fig. 1).

### Main outcome measure

All‐cause mortality during the observation period from 1 Jan 2008 to 31 Dec 2016 was determined through routine nationwide mortality tracing linked to the electronic health record. As previously described all previous and current SLAM contacts are checked monthly against the national mortality database and date of death is electronically flagged 2, 26.

### Follow‐up

For the clozapine group, the ZTAS ‘green’ blood result date was used as a proxy of the drug initiation. To be recognized as valid, clozapine has to be prescribed within 10 days of an initial green result being registered on the ZTAS. This provided the best marker for the date of clozapine initiation in the ZTAS database. For the non‐clozapine group, the drug initiation date was defined as the start date of the third antipsychotic use on hospitalization. For each patient, the follow‐up time ended at the end of the observation window (31 Dec 2016) or with the patient's death, whichever occurred first. The follow‐up period was restricted to maximum five years (1826.25 days).

### Other exposure variables

Age calculation was based on age at initiation of clozapine or the third novel antipsychotic. Multiple deprivation index was used as a proxy of socioeconomic status 27. This index was based on a subject's address group (area unit). Each area unit has its population of 1500 residents on average, with various unit‐level characteristics (employment, income, education, health, barriers to housing and services, crime, living environment) weighted according to the importance. In the present study, the level of multiple deprivation was divided into quartiles, and homeless and unknown were given a category separately.

As a measure of clinical monitoring, clinical contact days were calculated by summation of all in‐patient and out‐patient visits during the follow‐up, in which one in‐patient visit was counted as one contact day regardless of the duration of admission 2. Considering the varied follow‐up period in each subject, face‐to‐face clinical contact days were divided by individual follow‐up days, and then the percentage was categorized into quartiles.

The clozapine and non‐clozapine groups were meant to be clinically equivalent, but there is still a possibility of selection bias because overall health status can affect clozapine prescription. To address this issue, the present study used the Health of the Nation Outcome Scale (HoNOS) tool 28, 29, an instrument used by clinicians to monitor the health and wellbeing of psychiatric patients in the United Kingdom. The HoNOS data were extracted based on the date closest to the clozapine or 3^rd^ antipsychotic drug initiation date. HoNOS variables used in this study were divided into three categories: mental health symptom severity (overactive, aggressive, disruptive or agitated behaviour, hallucination and delusions and depressed mood), additional mental and physical health problems (non‐accidental self‐injury, problems of drinking or drug taking and physical illness or disability problems) and functional status (activities of daily living, standard of living conditions, occupational and recreational activities and social relationships). Every variable was categorized as no, minor, mild, moderate and severe problem. In addition, any ICD‐10 diagnoses F11 opiate and F10 alcohol use related disorders, recorded before or during the observation period, were also extracted from structured fields and free‐text records.

### Statistical analysis

All analyses were conducted using stata version 12 30. Chi‐squared tests were used to make comparisons between the clozapine and non‐clozapine groups. To estimate the risk of mortality due to clozapine use, Cox proportional hazard models were used, in which the clozapine group was coded as 1 and the non‐clozapine group as 0. Our initial multivariable model added sociodemographic variables as co‐variates (age, gender, ethnicity, marital status and deprivation level). We then added, cumulatively, a number of co‐variate sets as potential confounders. The sequence of adding these co‐variate sets followed the order used in a prior study examining clozapine exposure and mortality 2. These sets included a history of substance use disorders (alcohol and opioid), clinical monitoring (face‐to‐face contact days), mental health symptom severity (agitated behaviour, hallucination and delusions and depressed mood), additional mental and physical health problems (non‐accidental self‐injury, problems of drinking or drug taking and physical illness or disability problems) and functional status (Activities of daily living [ADLs] standard of living conditions, occupational and recreational activities and social relationships). Age was included in the Cox regression models as a continuous variable, and the remaining variables were treated as categorical variables. Hazard ratios (HRs) and 95% confidence intervals (95% CIs) were presented. Cox proportional hazard assumptions were checked via likelihood ratio tests to examine whether there was interaction with time. These tests were used to ensure that the relationship between clozapine and mortality did not change over time.

## Results

The mean [standard deviation (SD)] follow‐up time was 1413.8 (559.2) days. A total of 2837 TRS patients consisted of *n* = 1025 clozapine group and *n* = 1812 non‐clozapine group (Table 1). The mean age was 38.7 years (SD, 11.4) and men accounted for 62.5%. Characterized by mental health symptom severity, 41.8% of subjects had moderate or severe problems in hallucinations and delusions, whereas 6.1% in depressed mood. The proportion of deaths in subjects with moderate problem of physical illness was higher (13.2%) than those of other categories.

**Table 1:** Characteristics of study subjects (*N* = 2837)

	Total no. (% of total sample)	No. of deaths (% per characteristic)
Antipsychotic use		
Clozapine	1025 (36.1)	35 (3.4)
Other antipsychotics	1812 (63.9)	75 (4.1)
Sociodemographic variables		
Age (mean 38.7, standard deviation 11.4, range 17–71 years)		
<35 years	1176 (41.5)	28 (2.4)
35–<55 years	1406 (49.6)	55 (3.9)
55 years or older	255 (9.0)	27 (10.6)
Gender		
Women	1063 (37.5)	66 (3.7)
Men	1774 (62.5)	44 (4.1)
Ethnicity		
White	1003 (35.4)	52 (5.2)
Black	1434 (50.6)	44 (3.1)
Asian	158 (5.6)	8 (5.1)
Mixed	237 (8.4)	6 (2.5)
Other or not stated	5 (0.2)	0 (0.0)
Marital status		
Single	2327 (82.0)	93 (4.0)
Married or cohabiting	239 (8.4)	5 (2.1)
Divorced or separated	254 (9.0)	10 (3.9)
Not known or not disclosed	17 (0.6)	2 (11.8)
Deprivation level in area of residence		
Quartile 1 (the highest)	590 (20.8)	23 (3.9)
Quartile 2	592 (20.9)	23 (3.9)
Quartile 3	588 (20.7)	18 (3.1)
Quartile 4	601 (21.2)	25 (4.2)
Homeless	101 (3.6)	1 (1.0)
Unknown	365 (12.9)	20 (5.5)
Substance use disorders		
History of alcohol‐related disorders		
No	2679 (94.4)	99 (3.7)
Yes	158 (5.6)	11 (7.0)
History of opioid‐related disorders		
No	2778 (97.9)	108 (3.9)
Yes	59 (2.1)	2 (3.4)
Clinical monitoring		
Face‐to‐face contact days (% of follow‐up time)		
Quartile 1	709 (25.0)	40 (5.6)
Quartile 2	711 (25.1)	23 (3.2)
Quartile 3	708 (25.0)	23 (3.2)
Quartile 4 (the highest)	709 (25.0)	24 (3.4)
Mental health symptom severity		
Agitated behaviour		
No problem	1138 (40.1)	41 (3.6)
Minor problem	646 (22.8)	22 (3.4)
Mild problem	508 (17.9)	18 (3.5)
Moderate problem	312 (11.0)	23 (7.4)
Severe problem	188 (6.6)	4 (2.1)
Missing	45 (1.6)	2 (4.4)
Hallucinations and delusions		
No problem	511 (18.0)	15 (2.9)
Minor problem	378 (13.3)	12 (3.2)
Mild problem	718 (25.3)	30 (4.2)
Moderate problem	776 (27.4)	29 (3.7)
Severe problem	407 (14.4)	22 (5.4)
Missing	47 (1.7)	2 (4.3)
Depressed mood		
No problem	1330 (46.9)	42 (3.2)
Minor problem	822 (29.0)	33 (4.0)
Mild problem	466 (16.4)	24 (5.2)
Moderate problem	139 (4.9)	8 (5.8)
Severe problem	35 (1.2)	1 (2.9)
Missing	45 (1.6)	2 (4.4)
Additional mental and physical health problems		
Non‐accidental self‐injury		
No problem	2450 (86.4)	97 (4.0)
Minor problem	164 (5.8)	6 (3.7)
Mild problem	94 (3.3)	3 (3.2)
Moderate problem	54 (1.9)	2 (3.7)
Severe problem	29 (1.0)	0 (0.0)
Missing	46 (1.6)	2 (4.3)
Problem‐drinking or drug taking		
No problem	1930 (68.0)	78 (4.0)
Minor problem	286 (10.1)	12 (4.2)
Mild problem	285 (10.1)	10 (3.5)
Moderate problem	216 (7.6)	6 (2.8)
Severe problem	69 (2.4)	2 (2.9)
Missing	51 (1.8)	2 (3.9)
Physical illness or disability problems		
No problem	1824 (64.3)	53 (2.9)
Minor problem	459 (16.2)	14 (3.1)
Mild problem	341 (12.0)	21 (6.2)
Moderate problem	129 (4.6)	17 (13.2)
Severe problem	40 (1.4)	3 (7.5)
Missing	44 (1.6)	2 (4.5)
Functional status		
Activities of daily living (ADLs)		
No problem	1111 (39.2)	33 (3.0)
Minor problem	714 (25.2)	23 (3.2)
Mild problem	600 (21.2)	37 (6.2)
Moderate problem	299 (10.5)	13 (4.3)
Severe problem	66 (2.3)	2 (3.0)
Missing	47 (1.7)	2 (4.3)
Standard of living conditions		
No problem	1512 (53.3)	50 (3.3)
Minor problem	528 (18.6)	27 (5.1)
Mild problem	351 (12.4)	15 (4.3)
Moderate problem	181 (6.4)	7 (3.9)
Severe problem	175 (6.2)	6 (3.4)
Missing	90 (3.2)	5 (5.6)
Occupational and recreational activities		
No problem	1060 (37.4)	41 (3.9)
Minor problem	691 (24.4)	27 (3.9)
Mild problem	660 (23.3)	21 (3.2)
Moderate problem	237 (8.4)	14 (5.9)
Severe problem	102 (3.6)	3 (2.9)
Missing	87 (3.1)	4 (4.6)
Social relationships		
No problem	874 (30.8)	31 (3.5)
Minor problem	713 (25.1)	22 (3.1)
Mild problem	754 (26.6)	31 (4.1)
Moderate problem	331 (11.7)	18 (5.4)
Severe problem	117 (4.1)	5 (4.3)
Missing	48 (1.7)	3 (6.3)

When comparing non‐clozapine treatment‐resistant group with the clozapine group (Table 2), the clozapine group had more frequent face‐to‐face clinical contact (*P* < 0.001), but less severe baseline psychopathology profiles (*P* < 0.001) than the non‐clozapine group. The clozapine group had lower rates of substance use disorders, less severe symptoms of agitation, drinking and drug problems, better indices of living conditions and social relationships. The same tendency was observed when we restricted the analysis to the subset of participants with a HoNOS completed prior to clozapine initiation date (Table S1).

**Table 2:** Characteristics of clozapine and other antipsychotic groups in patients with treatment‐resistant schizophrenia

	Clozapine (*N* = 1025)	Non‐clozapine (*N* = 1812)	*P* a
Sociodemographic variables			
Age, mean (standard deviation)	39.2 (10.7)	38.4 (11.8)	
<35 years	385 (37.6)	791 (43.7)	<0.001
35 to <55 years	561 (54.7)	845 (46.6)	
55 years or older	79 (7.7)	176 (9.7)	
Gender			0.002
Women	679 (66.2)	1095 (60.4)	
Men	346 (33.8)	717 (39.6)	
Ethnicity			<0.001
White	456 (44.5)	547 (30.2)	
Black	422 (41.2)	1012 (55.8)	
Asian	61 (6.0)	97 (5.4)	
Mixed	84 (8.2)	153 (8.4)	
Other or not stated	2 (0.2)	3 (0.2)	
Marital status			0.084
Single	863 (84.2)	1464 (80.8)	
Married or cohabiting	82 (8.0)	157 (8.7)	
Divorced or separated	74 (7.2)	180 (9.9)	
Not known or not disclosed	6 (0.6)	11 (0.6)	
Deprivation level in area of residence			<0.001
Quartile 1 (the highest)	212 (20.7)	378 (20.9)	
Quartile 2	213 (20.8)	379 (20.9)	
Quartile 3	171 (16.7)	417 (23.0)	
Quartile 4	199 (19.4)	402 (22.2)	
Homeless	22 (2.1)	79 (4.4)	
Unknown	208 (20.3)	157 (8.7)	
Substance use disorders			
History of alcohol‐related disorders			0.059
No	979 (95.5)	1700 (93.8)	
Yes	46 (4.5)	112 (6.2)	
History of opioid‐related disorders			0.005
No	1014 (98.9)	1764 (97.4)	
Yes	11 (1.1)	48 (2.6)	
Clinical monitoring			
Face‐to‐face contact days (% of follow‐up time)			<0.001
Quartile 1	183 (17.9)	526 (29.0)	
Quartile 2	183 (17.9)	528 (29.1)	
Quartile 3	263 (25.7)	445 (24.6)	
Quartile 4 (the highest)	396 (38.6)	313 (17.3)	
Mental health symptom severity			
Agitated behaviour			<0.001
No problem	567 (55.3)	571 (31.5)	
Minor problem	220 (21.5)	426 (23.5)	
Mild problem	126 (12.3)	382 (21.1)	
Moderate problem	56 (5.5)	256 (14.1)	
Severe problem	34 (3.3)	154 (8.5)	
Missing	22 (2.1)	23 (1.3)	
Hallucinations and delusions			<0.001
No problem	218 (21.3)	293 (16.2)	
Minor problem	153 (14.9)	225 (12.4)	
Mild problem	291 (28.4)	427 (23.6)	
Moderate problem	227 (22.1)	549 (30.3)	
Severe problem	113 (11.0)	294 (16.2)	
Missing	23 (2.2)	24 (1.3)	
Depressed mood			0.002
No problem	495 (48.3)	835 (46.1)	
Minor problem	309 (30.1)	513 (28.3)	
Mild problem	159 (15.5)	307 (16.9)	
Moderate problem	31 (3.0)	108 (6.0)	
Severe problem	9 (0.9)	26 (1.4)	
Missing	22 (2.1)	23 (1.3)	
Additional mental and physical health problems			
Non‐accidental self‐injury			0.096
No problem	901 (87.9)	1549 (85.5)	
Minor problem	52 (5.1)	112 (6.2)	
Mild problem	27 (2.6)	67 (3.7)	
Moderate problem	15 (1.5)	39 (2.2)	
Severe problem	8 (0.8)	21 (1.2)	
Missing	22 (2.1)	24 (1.3)	
Problem‐drinking or drug taking			<0.001
No problem	752 (73.4)	1178 (65.0)	
Minor problem	105 (10.2)	181 (10.0)	
Mild problem	80 (7.8)	205 (11.3)	
Moderate problem	57 (5.6)	159 (8.8)	
Severe problem	6 (0.6)	63 (3.5)	
Missing	25 (2.4)	26 (1.4)	
Physical illness or disability problems			<0.001
No problem	596 (58.1)	1228 (67.8)	
Minor problem	196 (19.1)	263 (14.5)	
Mild problem	146 (14.2)	195 (10.8)	
Moderate problem	55 (5.4)	74 (4.1)	
Severe problem	10 (1.0)	30 (1.7)	
Missing	22 (2.1)	22 (1.2)	
Functional status			
Activities of daily living (ADLs)			0.219
No problem	402 (39.2)	709 (39.1)	
Minor problem	269 (26.2)	445 (24.6)	
Mild problem	210 (20.5)	390 (21.5)	
Moderate problem	103 (10.0)	196 (10.8)	
Severe problem	18 (1.8)	48 (2.6)	
Missing	23 (2.2)	24 (1.3)	
Standard of living conditions			<0.001
No problem	637 (62.1)	875 (48.3)	
Minor problem	186 (18.1)	342 (18.9)	
Mild problem	88 (8.6)	263 (14.5)	
Moderate problem	48 (4.7)	133 (7.3)	
Severe problem	28 (2.7)	147 (8.1)	
Missing	38 (3.7)	52 (2.9)	
Occupational and recreational activities			<0.001
No problem	433 (42.2)	627 (34.6)	
Minor problem	269 (26.2)	422 (23.3)	
Mild problem	195 (19.0)	465 (25.7)	
Moderate problem	68 (6.6)	169 (9.3)	
Severe problem	25 (2.4)	77 (4.2)	
Missing	35 (3.4)	52 (2.9)	
Social relationships			<0.001
No problem	358 (34.9)	516 (28.5)	
Minor problem	282 (27.5)	431 (23.8)	
Mild problem	234 (22.8)	520 (28.7)	
Moderate problem	97 (9.5)	234 (12.9)	
Severe problem	32 (3.1)	85 (4.7)	
Missing	22 (2.1)	26 (1.4)	

a *P*values represent the significance of difference between the clozapine and non‐clozapine groups, as assessed by chi‐squared tests for categorical variables and the student independent *t* test for continuous variables.

**Figure 2:**
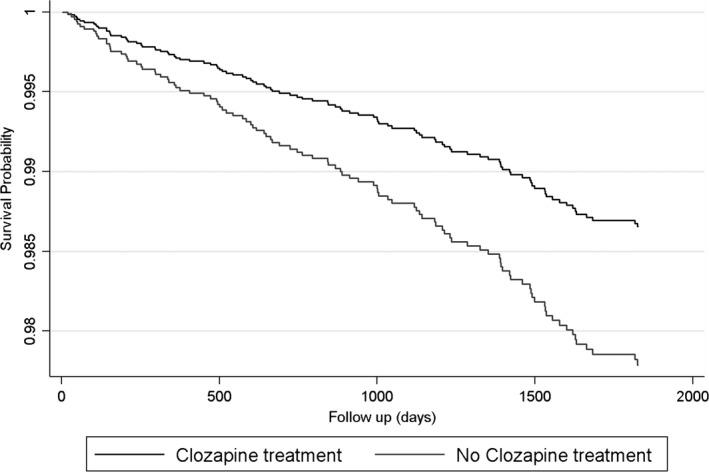
Survival curves of all‐cause mortality in patients with treatment‐resistant schizophrenia, stratified by antipsychotic use.

Likelihood‐ratio tests showed the proportional hazards assumptions held (*P* = 0.39). The protective effect of clozapine use on 5‐year mortality was not significant in the crude Cox regression model (HR, 0.73; 95% CI: 0.49–1.08; *P* = 0.12) (Table 3). Adjustment for sociodemographic variables (HR, 0.60; 95% CI: 0.40–0.92; *P* = 0.02) showed clozapine was significantly associated with a reduced mortality. Further adjustment for history of substance use disorders, clinical monitoring frequency, mental health symptom severity and additional mental and physical health problems (HR, 0.66; 95% CI: 0.42–1.03; *P* = 0.07) demonstrated a similar, but non‐significant effect – broadening confidence intervals suggest reduced power due to missing values reducing the available sample, rather than significant attenuation of effect. The final multivariable model, including all measured covariates, showed a statistically significant protective effect of clozapine on all‐cause mortality (HR, 0.61; 95% 0.38–0.97; *P* = 0.04). Fig. 2 provides the survival probability curves during follow‐up for the clozapine and non‐clozapine groups.

**Table 3:** Hazard ratios of all‐cause mortality related to clozapine use in patients with treatment‐resistant schizophrenia

	Deaths/total *N*	HRs (95% CIs)	*P*
Crude	110/2837	0.73 (0.49–1.08)	0.12
Multivariate Cox proportional hazard models adjusted for			
Sociodemographic variables	110/2837	0.60 (0.40–0.92)	0.02
Plus history of substance use disorders	110/2837	0.61 (0.40–0.93)	0.02
Plus clinical monitoring	110/2837	0.67 (0.43–1.03)	0.07
Plus mental health symptom severity	108/2788	0.71 (0.46–1.11)	0.14
Plus additional mental and physical health problems	108/2783	0.66 (0.42–1.03)	0.07
Plus functional status	104/2720	0.61 (0.38–0.97)	0.04

HR, hazard ratio; 95% CI, 95% confidence interval.

## Discussion

We found a protective effect of clozapine use on all‐cause mortality in TRS patients over a 5‐year follow‐up period. This is the first investigation to use electronic clinical notes (enhanced by linked data provided by a clozapine regulatory body) to describe the association of clozapine with all‐cause mortality in TRS. These data from a comprehensive specialist mental health care service within a geographic catchment, provided a greater depth of information to characterize patients with TRS than prior registry‐based studies 8, 31. We overcame the limitations of previous work by: restricting our analyses to TRS samples to reduce selection bias; taking account of differential baseline risks including psychopathology (hallucinations, delusions, aggression, subclinical depression, addiction) and functional status (problems with ADL impairment, occupational and recreational activities, social relationships, living conditions); and addressing potential impact of survival biases.

The direction of effect we found was consistent with a recent meta‐analysis, which reported significant pooled rate ratio of 0.34 in favour of patients using clozapine (95% CI = 0.19–0.62, *P* ≤ 0.001) comparing clozapine users to non‐users and/or other antipsychotic users 9. However, a previous study including 29 823 patients with schizophrenia in Sweden suggested that clozapine was not the most favourable antipsychotic in terms of reducing mortality, which may be attributable to more restricted clozapine use in Sweden or methodological difficulties access hospital prescribing data 25. Focusing on TRS patients, Stroup et al. 31 reported null findings, but they only followed their study subjects (*n* = 3123) for one year (explaining the relatively lower mortality rates across the total sample) and used United States Medicaid data, which has restricted coverage related to patients’ income and level of disability. Meanwhile, the Danish study (*n* = 2370) 8 used the national registry covering all individuals in Denmark. This study showed an adjusted HR of death related to non‐clozapine use of 1.88 (95% CI: 1.16–3.05), which equates to reduced risk of mortality in the clozapine group of 0.53, similar to the effect found in the present study (HR = 0.61).

Clozapine is associated with improved treatment outcomes in TRS patients 32, 33, 34 but polypharmacy or high doses of non‐clozapine antipsychotics predates clozapine treatment in around a third of treatment‐refractory patients, leading to delayed clozapine initiation 17. In the present study, we found 42% of the clozapine group had a possible delay in clozapine initiation (i.e., clozapine use after three or more attempts of non‐clozapine antipsychotics). Although beyond the scope of this study, future analyses should examine, within the clozapine subset, whether the delay between meeting TRS criteria and receiving clozapine is associated with greater mortality rates.

Prior naturalistic studies in treatment‐resistant populations have demonstrated that those not receiving clozapine often show characteristic differences from those receiving clozapine 9, 31. In our study, we found the clozapine group had high but less severe baseline psychopathology profiles than the non‐clozapine treatment‐resistant group in a number of domains, including less severe symptoms of agitation, drinking and drug problems, adverse living conditions and poor social relationships. These factors are unlikely to distort the protective effect we found of clozapine on mortality as they were accounted for in the analyses. However, these differences reveal the potential selection effects for clozapine initiation, and suggest certain phenotypic and environmental differences which may be behind clozapine treatment delay. It is possible that these selection effects are due to patient preference, but they also may be driven by health service rationing. Potentially, clinicians are being pragmatic and not attempting to trial certain groups of highly morbid patients for clozapine initiation.

Strengths of this study include restriction of the study population to patients with psychiatric treatment consistent with treatment resistance, adjustment for an array of potential demographic and clinical confounders, and the use of a comparison group receiving active treatment. Furthermore, the present study used data covering all mental health contacts in the catchment areas (community and hospital care), with timely linkage to accurate mortality data based on national registers. Specific to this study, the SLAM EHR including mortality data was linked to national ZTAS clozapine registry, which enabled us to improve identification of patients with clozapine initiation.

This study had a number of methodological limitations. Cause of death data was not available for analysis within our existing governance arrangements. Registrar reports on death dates are received by NHS organizations routinely from local registrars; however, cause of data death is administered separately by the Office National Statistics, and is only available through specific permissions, which has not been provided for this linked data study. We used an automated approach, similar to the previous study by Wimberley et al. 8, to identify those highly likely to meet TRS criteria and who have not been previously trialled on clozapine. This might lead to misclassification, possibly including non‐TRS patients in the analysis. However, misclassification is more likely to be non‐differential because the assignment of exposure status (prescribing clozapine) and recording of outcome status (mortality tracing) were independent of each other. There is still potential residual confounding by unmeasured confounders such as comorbid cardiovascular disease and smoking. Some previous studies found the protective effect of clozapine on all‐cause mortality after adjustment of baseline history of cardiovascular disease including ischaemic heart disease 4, 6. However, after clozapine initiation, the likelihood of having cardiometabolic illnesses such as hypertension, diabetes mellitus and hyperlipidaemia may increase 15, 35, 36. Because of the cardiovascular adverse effects of the drug, it is possible that clinicians may more strongly advise smoking cessation to clozapine users, with this leading to reduced smoking behaviour 37. That said an increased risk of cardiometabolic illnesses does not necessarily lead to death, and a previous study showed that smoking was not associated with the risk of cardiovascular deaths in 10‐year follow‐up of clozapine users 36. To obtain a valid estimation for the pure effect of clozapine on mortality, it would be useful to include healthy behaviours as time‐dependent variables in analyses. Dose information was not available in the current dataset, as suggested by Torniainen et al. 23, high doses of antipsychotics may be related to cardiovascular mortality; but low or moderate doses may not. Therefore, the reduced risk of mortality linked to clozapine observed in this study might be derived from the use of high‐dose non‐clozapine antipsychotics in TRS patients. Finally, a potential bias could have been introduced if the clozapine group had a greater number of extracted HoNOS scores from initial hospital treatment stage than the non‐clozapine groups. HoNOS scores tend to be higher in newly hospitalized samples than those taken at point of discharge or in the community. However, this potential bias is unlikely to have impacted the results, as we found no significant discrepancy in the distribution of treatment stages for extracted HONOS scores between clozapine and non‐clozapine groups.

There are several potential pathways for the protective effect of clozapine on mortality. It may be, from a whole clinical population perspective, that the benefits of clozapine's effectiveness in TRS at reducing psychopathology over other antipsychotics, outweighs its associated risk for later cardiometabolic disease 35, 36, 38. Certainly, reducing psychotic symptoms would provide more opportunity for patients to adopt healthier lifestyles such as reduced smoking and drinking 6. The effects of regular mandatory physical health screening cannot be completely excluded. The requirement for a patient's white blood cell and neutrophil to be within normal ranges in the United Kingdom, suggests that the clozapine TRS group may have a healthier blood profiles compared to the non‐clozapine group. However, abnormal blood profiles such as high leucocyte which increase the risks for cardiovascular events and all‐cause mortality 39, 40 does not exclude clozapine initiation. The intensity of clinical monitoring associated with clozapine use in TRS populations, may permit earlier detection and treatment of incidental physical health problems compared to non‐clozapine TRS groups; although Hayes et al.^2^ did take account of this increased clinical engagement and still found the protective effect of clozapine on mortality remained. Finally, it is possible that clozapine could enhance health by reducing the risk of inflammation‐related pathophysiology 41. Animal studies have provided consistent evidence on clozapine's anti‐inflammatory effects in the periphery, compared to other antipsychotics such as haloperidol, risperidone and quetiapine 42. Whether through changes in lifestyle or improving later immune/inflammatory responses in patients with TRS postclozapine initiation, further investigations are warranted to examine what factors mediate the observed protective effect of clozapine on mortality.

In summary, our findings suggest that in TRS, clozapine users have a reduced mortality risk compared to patients who have not received clozapine treatment. We add to the burgeoning evidence that suggests clozapine may help reduce health inequalities experienced by individuals with TRS, and ultimately improve their life‐expectancy 1, 9. Our findings support the argument for a re‐evaluation of clozapine treatment within clinical settings, especially for better resourcing services which aim to improve the initiation and continuation of clozapine for patients with TRS.

## Declaration of interest

RH has received research funding from Roche, Pfizer, Janssen and Lundbeck.

## Funding

This work was supported by the Clinical Records Interactive Search (CRIS) system funded and developed by the National Institute for Health Research (NIHR) Mental Health Biomedical Research Centre at South London and Maudsley NHS Foundation Trust and King's College London and a joint infrastructure grant from Guy's and St Thomas’ Charity and the Maudsley Charity (grant number BRC‐2011‐10035). JD was supported by a Medical Research Council (MRC) Clinical Research Training Fellowship (MR/L017105/1) and Psychiatry Research Trust Peggy Pollak Research Fellowship in Developmental Psychiatry. RDH was funded by a Medical Research Council (MRC) Population Health Scientist Fellowship (grant number MR/J01219X/1). GK, HS and RH have received salary support from the National Institute for Health Research (NIHR) Mental Health Biomedical Research Centre at South London and Maudsley NHS Foundation Trust and King's College London. The views expressed are those of the author(s) and not necessarily those of the NHS, the NIHR or the Department of Health. The above funding had no role in the study design; in the collection, analysis and interpretation of the data; in the writing of the report; and in the decision to submit the paper for publication.

## Supporting information


**Table S1**. Mental and physical health problems and functional status* of the clozapine and non‐clozapine groups in patients with treatment‐resistant schizophrenia
