# Supplementary material for: Clozapine and all‐cause mortality in treatment‐resistant schizophrenia: a historical cohort study
Source: Acta Psychiatr Scand. 2018 Dec 16;139(3):237–47. doi: 10.1111/acps.12989 (PMC6492259; doi:10.1111/acps.12989)
Supplement: Supplementary file 1 — Table S1. Mental and physical health problems and functional status* of the clozapine and non‐clozapine groups in patients with treatment‐resistant schizophrenia [file ACPS-139-237-s001.docx]

| **Supplementary Table. Mental and physical health problems and functional status^*^ of the clozapine and non-clozapine groups in patients with treatment-resistant schizophrenia** | | | | | |
| --- | --- | --- | --- | --- | --- |
|  | **Clozapine  (N=1025)** | | **Non-clozapine (N=1812)** | | **p**^†^ |
| ***Mental health symptom severity*** |  |  |  |  |  |
| Agitated behaviour |  |  |  |  | <0.001 |
| No problem | 363 | (52.5) | 511 | (31.2) |  |
| Minor problem | 147 | (21.3) | 391 | (23.8) |  |
| Mild problem | 101 | (14.6) | 357 | (21.8) |  |
| Moderate problem | 50 | (7.2) | 239 | (14.6) |  |
| Severe problem | 30 | (4.3) | 142 | (8.7) |  |
| Missing | 334 |  | 172 |  |  |
| Hallucinations and delusions |  |  |  |  | 0.007 |
| No problem | 98 | (14.2) | 254 | (15.6) |  |
| Minor problem | 92 | (13.3) | 210 | (12.9) |  |
| Mild problem | 207 | (30.0) | 374 | (22.9) |  |
| Moderate problem | 193 | (27.9) | 516 | (31.6) |  |
| Severe problem | 101 | (14.6) | 278 | (17.0) |  |
| Missing | 334 |  | 180 |  |  |
| Depressed mood |  |  |  |  | 0.085 |
| No problem | 306 | (44.3) | 748 | (45.9) |  |
| Minor problem | 216 | (31.3) | 470 | (28.9) |  |
| Mild problem | 135 | (19.6) | 287 | (17.6) |  |
| Moderate problem | 25 | (3.6) | 100 | (6.1) |  |
| Severe problem | 8 | (1.2) | 23 | (1.4) |  |
| Missing | 335 |  | 184 |  |  |
| ***Additional mental and physical health problems*** | |  |  |  |  |
| Non-accidental self-injury |  |  |  |  | 0.799 |
| No problem | 607 | (88.1) | 1416 | (86.6) |  |
| Minor problem | 39 | (5.7) | 101 | (6.2) |  |
| Mild problem | 24 | (3.5) | 61 | (3.7) |  |
| Moderate problem | 11 | (1.6) | 38 | (2.3) |  |
| Severe problem | 8 | (1.2) | 20 | (1.2) |  |
| Missing | 336 |  | 176 |  |  |
| Problem-drinking or drug taking |  |  |  |  | <0.001 |
| No problem | 503 | (73.5) | 1055 | (66.2) |  |
| Minor problem | 74 | (10.8) | 157 | (9.8) |  |
| Mild problem | 57 | (8.3) | 179 | (11.2) |  |
| Moderate problem | 45 | (6.6) | 148 | (9.3) |  |
| Severe problem | 5 | (0.7) | 55 | (3.5) |  |
| Missing | 341 |  | 218 |  |  |
| Physical illness or disability problems |  |  |  |  | 0.009 |
| No problem | 424 | (61.6) | 1125 | (68.9) |  |
| Minor problem | 125 | (18.2) | 239 | (14.6) |  |
| Mild problem | 94 | (13.7) | 176 | (10.8) |  |
| Moderate problem | 36 | (5.2) | 64 | (3.9) |  |
| Severe problem | 9 | (1.3) | 28 | (1.7) |  |
| Missing | 337 |  | 180 |  |  |
| ***Functional status*** |  |  |  |  |  |
| Activities of daily living (ADLs) |  |  |  |  | 0.617 |
| No problem | 258 | (37.5) | 647 | (40.0) |  |
| Minor problem | 187 | (27.2) | 395 | (24.4) |  |
| Mild problem | 153 | (22.2) | 349 | (21.6) |  |
| Moderate problem | 73 | (10.6) | 179 | (11.1) |  |
| Severe problem | 17 | (2.5) | 46 | (2.8) |  |
| Missing | 337 |  | 196 |  |  |
| Standard of living conditions |  |  |  |  | <0.001 |
| No problem | 399 | (60.5) | 743 | (49.0) |  |
| Minor problem | 128 | (19.4) | 291 | (19.2) |  |
| Mild problem | 73 | (11.1) | 228 | (15.0) |  |
| Moderate problem | 37 | (5.6) | 122 | (8.1) |  |
| Severe problem | 22 | (3.3) | 131 | (8.6) |  |
| Missing | 366 |  | 297 |  |  |
| Occupational and recreational activities |  |  |  |  | 0.005 |
| No problem | 279 | (41.8) | 550 | (36.0) |  |
| Minor problem | 170 | (25.5) | 355 | (23.2) |  |
| Mild problem | 151 | (22.6) | 405 | (26.5) |  |
| Moderate problem | 48 | (7.2) | 146 | (9.5) |  |
| Severe problem | 19 | (2.8) | 73 | (4.8) |  |
| Missing | 358 |  | 283 |  |  |
| Social relationships |  |  |  |  | 0.011 |
| No problem | 218 | (31.8) | 451 | (28.1) |  |
| Minor problem | 194 | (28.3) | 385 | (24.0) |  |
| Mild problem | 171 | (25.0) | 473 | (29.5) |  |
| Moderate problem | 74 | (10.8) | 219 | (13.6) |  |
| Severe problem | 28 | (4.1) | 78 | (4.9) |  |
| Missing | 340 |  | 206 |  |  |

^*^ Based on the information prior and closest to drug initiation date.

^†^ The significance of difference between the clozapine and non-clozapine groups, as assessed by chi-squared tests.
